# An evaluation of the implementation of cascade training for suicide prevention during the ‘Choose Life’ initiative in Scotland - utilizing Normalization Process Theory

**DOI:** 10.1186/s12913-019-4398-1

**Published:** 2019-08-20

**Authors:** Linda Gask, Nia Coupe, Gillian Green

**Affiliations:** 1 Centre for Primary Care, University of Manchester, Manchester, UK; 2 Manchester Centre for Health Psychology, University of Manchester, Manchester, UK; 3 STORM Skills Training CIC, Manchester, UK

**Keywords:** Education, Training, Cascade, Suicide-prevention, Implementation, Dissemination

## Abstract

**Background:**

‘Cascade training’ or ‘train-the-trainers’ has been widely utilised in the dissemination of information and expertise in health and social care, but with little examination of the work required for optimal delivery. National suicide prevention strategies commonly include such training initiatives.

**Methods:**

A qualitative study to *characterise the work,* according to the concepts of Normalization Process Theory, required to disseminate STORM, a model of suicide prevention training across Scotland, and then implement it within organisations. This utilised a cascade style ‘train-the trainers’ intervention delivered as part of the *Choose Life* suicide prevention strategy in Scotland during 2008–11. Semi-structured interviews were carried out with 19 training facilitators, 30 of their group participants within organisations and 11 local managers within health boards in Scotland.

**Results:**

Crucial to the process of a cascade training approach to implementing suicide prevention within an organisation was the multi-layered activity of constructing coherence of the intervention at every level in order to prevent dilution of the training. This necessitated collaborative work within and between groups of actors- managers, facilitators and participants. Where facilitators were effectively engaged in their role, confident in their ability to train, supported by supervision and possessed the leadership skills to engage both with participants and their local context to deliver training, there was evidence of both successful delivery and embedding within the organisation. However, there was little systematic evidence of institutional level appraisal- crucial to truly implementing a novel intervention within the system - despite efforts at local managerial engagement.

**Conclusions:**

Successful cascade or train-the-trainer implementation of an intervention requires extensive collaborative work to take place between and within groups of actors at all levels of an organization from those working at policy level to the ‘coalface’. A priori application of Normalization Process Theory, to specify aims and goals for the necessary work to be carried out between different groups of actors, would assist in embedding a novel working practice at all levels.

Future national training strategies for suicide prevention should address what is required to establish a flourishing culture of high-quality skills acquisition and development within healthcare organisations.

**Electronic supplementary material:**

The online version of this article (10.1186/s12913-019-4398-1) contains supplementary material, which is available to authorized users.

## Background

‘Cascade training’ has been widely utilised in the diffusion of information and expertise in health and social care, education, and industry. Within health and social care settings the method is more commonly referred to as ‘train the trainers’ (TTT). It is an approach that has been utilised, for example, in studies of the dissemination and implementation of new interventions to support care-givers after a stroke [1]; to train general practitioners in novel clinical skills for managing people with medically unexplained symptoms [2]; and in the implementation of other evidence-based clinical interventions [3–5]. It has also been employed to enable acquisition of the skills required to appraise the evidence base [6].

Cascade training can be defined as ‘*a series of training processes, each occurring as the result of the one before’* [7]. The term ‘cascade’ is meant to conjure the image of having information flow from one group to another until it reaches its final destination [8]. According to Jacobs [9], the first reported use of cascade training was to implement the job instruction training programmes as part of the Training Within Industry effort during the Second World War. The cascade method has been extensively employed internationally to train teachers in novel techniques.

In the cascade or ‘train the trainer’ process, it seems obvious that one group trains another group who then go on to train others and this has been seen as an inexpensive way of rapid dissemination. However, it is also a process in which the participants are both the subjects and the agents of change [10]. According to Jacobs [9] it is:

*‘the articulation of training programs to provide differing levels of competence they require to implement the change.’ … ‘thus the underlying notion of cascade training is that critical change related information will flow through the organisation in a planned way to facilitate subsequent parts of the institutionalization process.’ (p180)*

Training of trainers needs to ensure that those disseminating the training have acquired the knowledge, attitudes and skills - both to deliver the intervention *and* teach others how to do it. There is however the well-known problem that when information is re-transmitted to each level, the chances of dilution and or misinterpretation of key messages increase [4]. This has led to some considerable criticism of the effectiveness of the model in educational settings [11]. Indeed, in his educational blog Mackenzie [12], extends the water metaphor to explore a number of problems with the cascade model. These include the ‘sponge’ (skills and information not passed on by the second stage trainer) ‘trickle down’ (trainers not able to train participants to required standard) and the ‘flood’ (participants feel overwhelmed by what is delivered to them by trainers). McDevitt [13] commenting on training teachers in Botswana takes this further ‘*if you’re too far away from the source you can avoid getting soaked*’. He notes the conflict between involving teacher participation in modifying the input to meet local needs and ensuring the integrity of the message being transmitted to end users.

It is clear that attention should be paid, in the initial planning stages of a TTT programme, to the situation in which the training trainers will take place and the intervention will eventually be applied [14]. Indeed, there is increasing recognition of the role played by contextual factors including organisational history, management attitudes, team relationships, external policy and other service development issues. Bax [15] has powerfully argued the need to not merely understand the target social and cultural context but to:

*‘find strategies to develop awareness in the trainees themselves of their own context and to derive the context and framework of the development sessions from the trainees themselves.’* (p174)

Contextual factors have posed problems when the focus has primarily been on the implementation of the intervention at the professional-patient-carer level [1]. Yet detailed reflections on the limitations of cascade methodology is harder to locate in health and social care settings than in education, *despite* its widespread application in dissemination. A systematic review of train-the-trainers programmes (TTT) in health and social care [16] found that the heterogeneity of the studies and limited data prevented meta-analysis although narrative review found that the TTT programs in 13 studies helped to increase knowledge, improve clinical behaviour, or produce better patient outcomes. The authors of this review concluded that a ‘blended learning’ approach, combining different techniques, was most likely to be successful in achieving positive outcomes but failed to explore processes in detail. However three studies in this review identified a potential long-term problem with the model. It was often difficult to ensure the continuing implementation of the training programs due to high staff turnover and retention of staff after they had been trained. Therefore, long-term sustainability and staff commitment need to be considered when developing TTT programmes.

### STORM skills training and suicide prevention

STORM training (Skills Training On Risk Management) is a widely disseminated course in suicide prevention skills, designed primarily for clinical staff, which has been demonstrated to lead to both attitude change and acquisition of talking skills [17, 18]. National suicide prevention strategies commonly include training of health and social care professionals in suicide risk assessment and management [19–22] and STORM training was implemented across Scotland for clinical staff as one of a suite of training packages in response to the Scottish Government Choose Life initiative. This was a multifaceted programme launched in 2006 in an attempt to reduce the suicide rate in Scotland, which, at that time, was higher than elsewhere in the UK [23, 24]. Choose Life included the explicit aim of ensuring that *‘more than 50% of frontline NHS staff had received at least one specific course on suicide intervention’* (http://www.chooselife.net/Training/index.aspx). The four approaches employed by Choose Life were *STORM* (the subject of the present study and specifically aimed at front line healthcare staff*)*, Applied Suicide Intervention Skills training (*ASIST)* (https://www.livingworks.net/asist/)*,* Suicide Alertness for Everyone (*safeTALK)* (https://www.livingworks.net/safetalk/) and Mental Health First Aid (*SMHFA*) [25]. TTT programmes have been widely employed internationally to implement these and other suicide prevention training initiatives. Suicide prevention is a global public health priority, with a need for acquisition of skills throughout health and community services, not only specialist mental health care [26]. It thus requires training to be both widely disseminated across systems and locally implemented.

### Researching implementation

Introducing and embedding a new way of working into an organization requires a clear understanding of exactly what behaviours and practices need to change, what systems need to be put in place and what resources called upon, to support implementation. Numerous theories, models and frameworks have been described for understanding how and why implementation succeeds or fails [27].

In this study we have utilised Normalization Process Theory (NPT) [28], because it focuses primarily on exactly what needs to be done to change practice, and explicitly examines how interventions are adopted, embedded, and integrated into organizational routines. NPT is concerned with explaining what people do, rather than attitudes or beliefs and as such is an ‘Action’ theory. NPT proposes 4 different constructs, representing types of work that people do when implementing a new practice or practices in everyday work: Coherence, Cognitive Participation, Collective Action and Reflexive Monitoring. ‘Coherence’, is the ‘sense-making’ work that people do both individually and collectively when faced with the problem of operationalizing a set of novel practices: ‘*what is it that we have to do*?’ Like all NPT constructs it has 4 components which are helpfully summarised in the NPT toolkit (http://www.normalizationprocess.org) (see Table 1). ‘Cognitive Participation’ refers to the **work** that people do with each other to build and sustain a community of practice around a new technology or complex intervention: ‘*How can we make this happen*?’ ‘Collective Action’ is the operational work that people do to get new practices set up and embedded within the organisation-:

*‘What do we have to do at an organisational level to normalise this change*?’ And finally, ‘Reflexive Monitoring’ is the work that people do individually or together with others to appraise the impact of the new practices they have already been engaged in implementing: *‘What impact has this intervention had?’*

**Table 1:** The 16 questions for thinking through an implementation problem by application of Normalization Process Theory

Coherence	Cognitive participation	Collective Action	Reflexive Monitoring
Participants distinguish the intervention from current ways of working	Key individuals drive the intervention forward	Participants perform the task required by the intervention	Participants access information about the effects of the intervention
Participants collectively agree about the purpose of the intervention	Participants agree that the intervention should be part of their work	Participants maintain their trust in each other’s work and expertise through the intervention	Participants collectively assess the intervention as worthwhile
Participants individually understand what the intervention requires of them	Participants buy into the intervention	The work of the intervention is appropriately allocated to Participants	Participants individually assess the intervention as worthwhile
Participants construct the potential value of the intervention for their work.	Participants continue to support the intervention	The intervention is adequately supported by its host organization	Participants modify their work in response to their appraisal of the intervention

Our aim in this study was to explore exactly what work needs to be carried out in order to optimise the delivery of a cascade training intervention (also called TTT) to nationally disseminate, implement and embed an intervention in local organizations. We addressed this using an evaluation of the implementation of STORM suicide prevention training in Scotland, using NPT as our theoretical model.

## Methods

### Design

The study employed a qualitative design. Semi-structured interviews were carried out with a wide range of key informants (see below).

### Setting

The intervention was disseminated across and implemented within Scottish Health Boards during the Choose Life suicide prevention policy initiative.

***Ethical approval*** was obtained from Coventry and Warwickshire REC approval number 08/H1211/124.

### Intervention

Facilitators were trained by pre-existing STORM Consultant Facilitators to deliver STORM training within their local Health Boards across Scotland. Individuals were selected for facilitator training by Health Boards in conjunction with *Choose Life.* STORM training consists of up to 4 educational modules which can be delivered flexibly (but usually over 2–3 h each). These modules are: assessment, safety planning (called ‘crisis management’ at the time of training), problem solving and future safety planning (called crisis prevention). Training comprises brief lectures, video demonstration and discussion, role-rehearsal and video-feedback to acquire new skills. Facilitators then trained local groups of participants. Training for facilitators is carried out over 2 days, following a 2-day experience of being trained in the intervention (total duration of 4 days). The primary implementation strategy [29] employed in this study was training local facilitators (trainers) from organisations in group settings across Scotland. Local facilitators, who were also provided with materials (training slides and a manual), were encouraged to tailor training to local needs whilst retaining the elements considered essential to acquiring new clinical skills (role-play and videofeedback). They were also given the option of contact for supervision and support from the senior trainer (GG). A Template for Intervention Description and Replication (TIDieR) [30] checklist has been included in Additional file 1.

### Participants

Between 2008 and 2011, 97 subjects were trained as STORM facilitators. These in turn trained 1155 participants in the intervention, either individually or in pairs- facilitating 148 group sessions in suitable venues across Scotland. Of these sessions, 18 covered only 2 of the 4 modules. Of those trained, all the facilitators and 568 participants were agreeable to contact for interview by the research team.

Qualitative semi-structured telephone interviews were carried out by NC between September 2010 and November 2011 after attempting to contact again those who had indicated agreement to be approached at a later date. Some 60 individuals from 3 different groups were successfully recruited- 19 of the facilitators and 30 participants who all received training after April 2009. Additionally, 11 managers (who were either contacted through facilitators or recruited as either facilitators or participants but were also in a managerial position within the healthcare system or an individual organisation) were also recruited. All Health Boards except for NHS Borders, Orkney, Shetland and the Western Isles were represented across all three groups of Participants. See Table 2 for details of role and place of work of interviewees. Almost all those interviewed who were participants in STORM sessions had no experience of any other suicide prevention training beyond their basic professional training (3 ASIST; 1 SafeTALK; 26 no other training).

**Table 2:** Role and place of work: participants, facilitators and managers

Job/role		Health Board	
Facilitators (*n* = 19)			
CPN Service development officer Staff nurse Project nurse Nurse trainer Lecturer in mental health nursing Parasuicide nurse specialist Public health nurse Practice development nurse Nurse consultant Suicide prevention trainer Nurse therapist Clinical nurse specialist Deputy ward manager Senior CE practitioner Senior Nurse practitioner Care home education facilitator	1 1 1 1 2 1 1 1 1 1 2 1 1 1 1 1 1	Grampian Lothian Ayrshire & Arran (one private sector) Greater Glasgow and Clyde Dumfries & Galloway Lanarkshire Fife Highland	2 4 3 5 1 2 1 1
Managers (*n* = 11)			
Clinical nurse manager Clinical governance manager Team leader Programmes manager Choose Life Coordinator Head Occupational Therapist Training Coordinator Senior Lecturer Community Psychiatric Nurse	1 1 1 1 2 1 1 1 2	Grampian Lothian Ayrshire & Arran Greater Glasgow and Clyde Dumfries & Galloway Lanarkshire Fife Highland	1 4 1 0 1 0 2 2
Participants (*n* = 30)			
Community Psychiatric Nurse Staff Nurse Ward Manager GP Clinical Psychologist Occupational Therapist OT technician Chaplain Health Visitor Memory clinic nurse Support Worker Blood borne virus nurse	8 6 1 3 3 3 1 1 1 1 1 1	Grampian Lothian Ayrshire & Arran Greater Glasgow and Clyde Dumfries & Galloway Lanarkshire Fife Highland	5 10 1 1 2 1 4 6

Facilitators and managers were asked how they felt STORM had been received (into practice, policy and culture) and whether they were aware of any changes that had been made (in practice, policy and culture) as a direct consequence of the implementation process. Additionally, were they aware of any concerns or issues relating to the delivery and/or translation of STORM? Did they feel the strategies employed to implement STORM were appropriate and adequate? Did they think training was being offered to the right people/groups within the organisation? Managers were also asked what specific part they played in the implementation of the training and within the organisation. Interviews with participants, who received training in the intervention from facilitators, focused on their experience of and views about the training and the impact on their everyday practice- and this question was also asked of the facilitators themselves who were also practicing clinicians.

Data analysis was led by LG who coded the data utilizing a simple template [31] or ‘a priori’ coding manual specifically derived from May and colleague’s operationalization of the elements of NPT- the ‘toolkit’ (http://www.normalizationprocess.org) (see Table 1) to address implementation of a cascade model of training. This was then entered onto MAXqda qualitative analysis software. A total of 60 transcripts were included in the analysis. Only data that could be coded according to the NPT-derived template was considered -this covered all important points. The findings were discussed in detail between the authors and underwent subsequent revisions to achieve consensus. Every attempt was made to ensure that they accurately reflected the original data and the lessons to be learned from the study for future implementation.

## Results

In describing the work of disseminating the cascade training intervention for STORM, and implementing STORM within the organizations, we will consider the work carried out within and between groups of actors. A summary of the postulated work required by each group of actors to deliver the intervention, according to the elements of NPT, and which emerged from this analysis, can be found in Table 3 (with specific questions/tasks to characterise each stage in Table 1).

**Table 3:** the work required for implementation of the STORM cascade training intervention according to the concepts of NPT

	Coherence^a^	Cognitive^a^ participation	Action^a^	Reflexive monitoring^a^
Consultant trainers and training organisation	Work with facilitators and *each other* to construct coherent vision of training for this organisation	Training the facilitators	Enabling the facilitators to put training into action, support and supervision	Providing feedback to policy level commissioners. Modify the training in response to feedback from facilitators, participants and managers.
Senior managers	Work with policy level actors, other managers *and peers* to understand why and how to prioritize training	Ensure that training is taking place in the organisation	Ensure that training is adequately supported and being delivered optimally.	Evaluate and synthesize feedback from managers, facilitators, participants and support agencies. Provide feedback to policy level/ commissioners. Consider if/when/how to prioritize embedding within organisation.
Clinical managers	Work with senior managers, *fellow managers* facilitators, participants and support agencies to understand how and why to prioritize training	Ensure that conditions are optimal for training to take place in the organisation	Ensure that the right people are being trained, training is taking place, and being optimally supported	Provide feedback to senior managers. Obtain and synthesize feedback Negotiate with facilitators and senior managers what system changes required to embed within organisation.
Facilitators	Work with managers, fellow facilitators, participants and support agencies to understand how and why to prioritize training	Deliver the training to participants	Ensure that training is being optimally delivered	Provide feedback to managers and training organisation. Negotiate with clinical managers about what system changes required to embed within organisation. Negotiate requirements to normalize on-going training.

^a^See Table 1 for specific questions to be addressed/work to be carried out

### Co-constructing ‘coherence’: the ‘making sense’ work done between facilitators, participants and managers

By the end of their own training, some facilitators were able to construct a coherent vision of the value of the STORM training:

*‘STORM is central to the whole process. What STORM has given to us is a common language, it’s also … erm.. given us a framework … Now what that’s given us is uniformity and structure so if someone, you know, presented with very low suicidal intent … but in the past we didn’t have any record of what intervention had happened … so it’s given us structure to practice … and for me that will shape local practice.’* (Facilitator-59)

This was reflected in positive views of the need for training from many staff regardless of their previous access to training. Thus, an essential first hurdle in achieving a coherent view of the task was the success of the work carried out *between* the consultant trainers and facilitators in constructing a sense of ‘coherence’: understanding how STORM training was different from normal practice, why it was needed, what it required of them, and its particular value to the organisation. This might be more difficult if the person was initially ‘allocated’ to do the work of facilitation by management:

*‘to be honest with you I didn’t particularly want to do it because I had a very full and busy diary of other training that I’m looking at providing, I was sent because my manager knows I can deliver training’* (Facilitator-90)

This lack of engagement could then be reflected in their own difficulty *in turn* to work with their own respective course attendees in the tasks of co-constructing a coherent vision of STORM training with participants. Only 25 of the 97 facilitators who were trained actually delivered any training during the project lifetime. Training requires facilitators, as noted earlier, to persuade participants, (some of whom were also allocated without considering if this was the most suitable training for their role in the organisation), to engage in videorecorded role-play and videofeedback of their consultations. Unsuprisingly, this seemed more challenging for those facilitators whose own initial level of engagement and views of STORM were less than optimal.

Engaging participants could be challenging. Some did not perceive how the STORM approach to their consultations differed from their routine practice-

‘*it’s just part of the bread and butter of our everyday work really’* (Participant 681) and there was some dissent about whether preventing suicide was always possible or even desired.

*‘Although we have a duty of care obviously to people to try and stop them from harming themselves, I think it’s very much a personal choice thing and I can see where people would be … just be fully justified to say, no I’ve had enough.’* (Participant-893)

However, it wasn’t only course participants who had to be engaged in the work of constructing coherence. Managers of services also had to understand what the training was about and who it was potentially best aimed at, so facilitators had to work both ‘up’ and ‘down’ the cascade:

*‘It’s just targeting the right people, getting managers on board to see how it would be useful for the staff, but also the staff themselves in relation to being videoed’* (Facilitator-04)

Local support services had to be convinced of the need for suitable venues, adequate resources and provision of administrative support. Local managers had to engage their boards in understanding the importance of and need for training:

‘*really just get the board on side that this is something that we have to do … it seems to be at the right level now, people are all aware now so it’s good for us at the clinical level if we’ve got that buy-in and they understand because obviously we’ve got to backfill places …* ’ (Manager-01)

A key lever was the national HEAT (Health –Efficiency-Access- Treatment) target to train 50% of key front life staff in the NHS in suicide prevention by 2010 [23]. This effectively made training mandatory for the period that the target was in place. Support and supervision for facilitators was also offered by STORM and in some places local support for facilitators was provided by the Choose Life coordinator - both of these were perceived as helpful.

### Cognitive participation: working together to deliver the STORM training

As the facilitators gained experience of delivering the training, they developed ways of overcoming the difficulties in setting up and managing the equipment, getting participants to role play consultations and to undergo filming and videofeedback.

*‘Initially when you have that huge learning of delivery … IT … you know some of the equipment … once you overcome that, you find with some of the new recruits it takes them a while to feel competent and confident but when you’re delivering the suicide agenda, it can have an impact on yourself, given the nature of the subject. I think supervision’s essential’. (*Facilitator-59)

Enthusiastic facilitators engaged in the tasks of cognitive participation by actively getting on with the work of training. They found ways of managing unwillingness to engage in role-play or be recorded:

*‘ … before they come on the training , they know exactly what is expected of them, if they’re there at the training the expectation is that they will participate, if they’re having difficulties we say right from the beginning … speak to one of the facilitators … but if people don’t participate in all of the components of the training including the video people will not be certificated*.’ (Facilitator-66)

Facilitators gained confidence and reassurance from working in pairs and seeking supervision to manage problems:

*‘We do always get people who don’t want to do the training, and we’ve found when people haven’t read the pre-course information, it kind of comes as a real shock to them and then having to do that immediately, and I think we’re very much on top of working with that now, but initially it was ‘I don’t have to do that and if you make me do the filming you’re infringing my human rights’, and actually I got … myself and my colleague did ring up for some supervision from Gill and talked that over, that was very helpful.’* (Facilitator 48)

Groups where members did not usually work together tended to promote mutual learning better than those when whole clinical teams attended together, where awareness of pre-existing hierarchies might stifle discussion. However, team attendance did allow more consideration of translation into practice. Leadership was important at all levels- with some of the facilitators forging ahead in a local leadership role and certain key individuals taking this role at organisation level such as directors of public health, and participants at team level:

‘*I thought it might be useful for the team, so I asked to go on it’* (Participant-583).

Managerial support also assisted greatly with this:

*‘Quite a few people voiced the opinion that, you know, if you’ve been qualified for quite some time maybe it won’t be as helpful, but I think the manager was very much ‘well, we’ll go along and see.’* (Participant-681)

There was disappointment that hospital consultants didn’t fully participate:

*‘they just said they didn’t want to be videoed so just lasted half a day’* (Participant 681).

However, as implementation gained momentum, anxiety began to dissipate and take up of places increased with more demand for training.

### Collective action: getting the elements of the STORM intervention ‘normalised’ at all levels within the organisations

As noted above, the appropriate allocation of people to the roles of facilitator and participants (some of whom went on to become facilitators themselves) within the organisation was crucial. Some expressed concerns that STORM training was being offered to staff for whom it was not clinically required and that this constituted a ‘waste of resources’. Others approached this systematically:

*‘When I was identified as somebody who might like to do the training for trainers, I went along, completed that, sat down with my line manager … and we decided who we were going to target for STORM training … and then, because I coordinate training as part of my job in the hospital I just set about coordinating that. I work in in-patient units and we just teamed up together and we trained across community and in-patients’*. (Facilitator-27)

Organisational support was however variable. One facilitator noted how his host organisation had ‘*allowed us to train’* but it was clear that staffing numbers on wards might impact considerably at the last minute, and the quality of training venues varied from ‘a cupboard in an office’ and ‘an old ECT (Electroconvulsive Therapy) suite’ to the ‘absolutely fantastic’. Some of the facilitators were employed part-time specifically to train whereas others depended on the goodwill and interest of managers to be able to find time to release them to do training. When training was mandatory it was possible to provide it during working hours, but towards the end of the project this was changing and there was concern about whether participants would be prepared to attend in days off or pay for training.

Local managers were key in enabling those who had attended the training to translate what they had learned into facilitating change in both clinical behaviour and policies.

*‘In terms of translation into practice it’s quite difficult. All we can do really is attend meetings, so we attend meetings with our local clinical managers, and we will try and influence how STORM is translated into practice, and that’s possibly, that’s as far as we can go we try and make it a strong influence but ultimately it’s the decision of those clinical managers, so however much they take that forward.’* (Facilitator-48)

Nevertheless, one facilitator reported that managerial engagement had led to development of a new suicide prevention and treatment pathway utilizing constructs from the STORM training. The intervention was also successfully incorporated by another into an undergraduate nursing course that he was involved with locally (Facilitator-48).

### Reflexive monitoring: evaluating the the impact of the STORM training intervention

Individually, both facilitators and participants noted the mostly positive impact of the intervention on their clinical practice:

*‘I think it’s fitted in very well actually, yeah, yeah, I think that … erm … quite often people have kind of hinted at suicide before and maybe I haven’t always taken that seriously, and I think that I’m certainly more likely now to take it seriously or to look for reasons to take it seriously rather than think ‘well thank goodness, you don’t really mean that’, and I’m certainly not worried now about being very open and saying to people ‘have you ever thought of killing yourself?’, whereas before it would’ve been couched in euphemism’.* (Participant 702)

*‘it used to be that I felt that I couldn’t quite ask the death question, but now I find a way to ask that’* (Participant 621)

Some of the more experienced group participants felt that training was best aimed at younger people, a view that was not necessarily shared by the facilitators. However, where participants reported positive experiences of STORM training facilitators described how this seemed to create an internal ‘feedback loop’ within organisations- leading to increased work of coherence, cognitive participation and collective action and more demand for training:

*‘I think It’s been very positive, certainly the verbal feedback would suggest that, the fact that we have people who are asking for the training, and continuing to ask for the training, and we have people asking for updates, you know, ‘what happens now?”* (Facilitator-27)

However, there was an awareness of the potential problems of balancing the need to make the content of training more flexible to engage particular groups against maintaining the coherence and quality of STORM training:

*‘I could see lots of opportunities … but because you’re kind of confined in terms of rolling it out as a programme we can’t do that.’* (Facilitator-72)

*‘I think one of the problems is that I’ve found that [indistinct] quality of recent training compared to the training I did some time back … you know … there seems to be some adjustments … approaches that differ slightly, and I just wonder how trainers … you know for example … ensure that … you know … they are still meeting the mark and hitting the spot of STORM development.’* (Facilitator-59)

There was little evidence of systematic local collective monitoring within organisations - some limited oversight took place- mostly to assess performance against training targets:

*‘we do rely on reports from attendees to make sure that we’re getting the right people through, so those that are supposed to be attending to make sure we’re reaching the targets that we have to reach, because it’s the only way that we can cross reference that’* (Manager-01)

However, there was no clear evidence that this was being utilised to contribute to any longer-term planning to embed suicide prevention training expertise and culture within local health boards.

## Discussion

The training of nurses, doctors, allied health and social care professionals and other front staff is, internationally, one of the planks of suicide prevention policies - despite the absence of good quality evidence from randomized controlled trials for its effectiveness [20]. No randomized controlled trial (RCT) has succeeded in demonstrating that gatekeeper training alone affected suicide rates, and training is usually implemented along with other initiatives as part of a multifaceted model. This makes it difficult to identify the effect of this specific intervention on suicide rates*.* In the absence of such evidence, but in the knowledge that the availability of *personalized risk management without the use of checklists* does appear to be one of the key characteristics of safer mental health care [32], we continue to deliberate how best to deliver and disseminate the training required to provide this within organisations [19–22]. Cascade training models are a very common approach to delivery of training across healthcare. How can we *maximise* their potential impact to avoid the need in the future for repeated and potentially costly re-commissioning?

Crucial to the process of *implementing* STORM suicide prevention training within NHS boards in Scotland was the multi-layered activity of constructing coherence of the intervention at every level. By which we mean understanding the purpose, value and processes of the training intervention and the work each group of actors had to do to embed this in the organisation. For STORM this necessitated collaborative work across several interfaces: between consultant trainers and facilitators; facilitators and participants; facilitators and management; managers and board members, as well as within each of these groups- and ultimately- through a feedback loop, with participants who had benefited positively from training persuading management and colleagues of the value of it. The enactment of training in the organisation was underpinned by this essential foundation work- which was considerably more complex than envisaged by a simple ‘cascade’. To return to the water metaphors applied earlier [12], cascade training needs to be more akin to a series of superimposed ‘whirlpools’ rather than a one-way torrent of disseminated learning if it is to be effective. We suggest that this multi-layered activity of constructing coherence is not only important in TTT interventions but is an activity which seems key to implementing psychosocial interventions in several healthcare areas.

Where facilitators were effectively engaged in their role, confident in their ability to train, felt supported and possessed the leadership skills to engage both with participants and their local context to deliver training, there was evidence of both successful delivery and embedding within the organisation. However, there was little systematic evidence of reflexive monitoring. This is the work that ultimately defines and organises the everyday understanding of the intervention for the organisation such that there is formal appraisal *both by individuals and within the organisation* of the utility of the innovation. There was also concern, commonly expressed in evaluations of cascade models, about the dilution of the quality of the training over time and about the degree to which it could be modified to match the needs of the particular group being trained. STORM is a relatively plastic intervention in that its format can be modified to suit the context- however some trainers seemed unaware of or uncertain about the extent to which this was allowable.

Institutional level appraisal- an examination of whether reconfiguration of working practices actually had an impact on processes and outcomes of care was much more difficult to achieve, despite efforts at local managerial engagement. Monitoring and feedback of outcome data can be important in motivating professionals to change their behaviour [33]. In terms of suicide prevention we acknowledge that changes in outcomes can be very difficult to demonstrate reliably in terms of meaningful changes in attempted or completed suicide [20]. Nevertheless there was little evidence of any real evaluation by organisations of the delivery, acceptability and either quality of the training that was taking place or its subsequent impact on processes and quality of care. Such appraisal would have been very valuable in informing, and improving, through a feedback loop, the way in which training was being implemented.

Finally, in this study we noted that only a quarter of those trained as facilitators actually completed any further training of participants. Our findings suggest that the complex nature of ensuring implementation *within* the sites may have contributed to this.

Clarke and colleagues [1], utilizing a cascade model, noted that NPT constructs were helpful in identifying problematic processes in embedding a complex intervention using a cascade approach, but that NPT:

‘*tends to place undue emphasis on individual and collective agency without locating this within, and as shaped by, the organisational and relational context in which implementation occurs’*.

May and colleagues have in fact since described in some detail the processes involved in embedding healthcare innovations in practice from the perspective of NPT [34] and the work that actors do when they realize and execute interventions in organisational and relational settings [35]. The novel intervention itself to be enacted between participant and patient is only one aspect of the work- yet the one which is generally given prominence. However, at each of the above interfaces the work of implementing the intervention has to be coherently defined, enacted, embedded and monitored. Contexts are dynamic and negotiated. NPT proved extremely useful in this study in characterising exactly what work needs to carried out in the context of a dynamic work environment.

To return to the question posed above, Cascade or TTT models must address, as part of the training, the problems and potential solutions likely to be faced in implementation. Indeed, what might be most helpful when disseminating a cascade training intervention would be to define a priori what exactly is the work that needs to be carried out at *each interface*, within the organisation, and then keep this under regular review. This might be initially mapped out during a session on the training course then developed by a small steering group within the organisation. Further programmed support and supervision may also be important. Where the offer of supervision was taken up, this was helpful.

We also consider (although it was not specifically addressed in this study as policy makers were not interviewed) that policy may be an important lever here. In our view, much more policy work needs to be focused on promotion of *embedding change* rather than simply initiating it. Interpretation and dissemination of the policy of improving the skills of the workforce needed to be subject to a greater level of higher level institutional ‘coherence work’ at the stage of planning the intervention beyond the simple goal of *‘more than 50% of frontline NHS staff had received at least one specific course on suicide intervention’*. Guidance might be provided on how organisations should locally evaluate the impact of such training. For future research into wide-scale training initiatives of this kind we suggest the following questions should be posed in developing and evaluating the intervention. How can participants learn from the implementation process and ensure that there is an on-going plan to maintain and embed the intervention rather than simply switch to the next national target when it arrives? (A concern expressed in this study). How can the national policy target be built upon such that the inevitable loss of skill set within an institution over time, as experienced staff move on, be mitigated against? For those who are trained return to work in systems where nothing really changes, such a target may eventually be perceived as little more than a ‘*triumphalist symbolic action’* [13] However, as Johnson and May [35] note *‘successful interventions seem to restructure and reinforce new practice norms and associate them with peer and reference group behaviours (*p12) thus achieving a deeper level of both attitudinal and structural change within organisations that, in our previous experience, often lack an established culture of such training [18].

This study has a number of limitations. It was a pragmatic study of a ‘real-life’ policy implementation conducted nationally but not all health boards participated fully in training, so the findings potentially reflect a more positive view than they would otherwise. No observational data was collected and although efforts were made to interview as many facilitators and participants as possible, resources were limited, and only a relatively small proportion of those who both those participated in the training and managerial staff could be contacted successfully to take part. No data was collected from policy makers, senior managers, support services, service users or user groups therefore the potentially beneficial role of service user groups in facilitating implementation of such an intervention could not be examined. The evaluation was carried out by researchers who have been engaged in developing and disseminating STORM (see competing interests statement below) and there is a need for further independent evaluations of STORM.

## Conclusion

Successful cascade or train-the-trainer implementation of an intervention within an organisation requires extensive collaborative work to take place between and within groups at all levels, from those working at policy level to the ‘coalface’. Of particular importance to this, and the embedding of psychosocial interventions in healthcare and other settings, is the multi-layered activity of constructing coherence. A priori application of Normalization Process Theory, to specify aims and goals for the work to be carried out at all levels, might assist an organisation in achieving greater success in embedding novel working practices.

Future national training strategy for suicide prevention should consider how to not only disseminate and implement training within organisations but what is required to establish a flourishing culture of high-quality skills acquisition and development within healthcare organisations.

## Additional file

Additional file 1: Template for Intervention Description and Replication. (PDF 193 kb)

## Data Availability

The datasets used and analysed during the current study are available from the corresponding author on reasonable request.

## Abbreviations

ASIST: Applied Suicide Intervention Skills Training
HEAT: Health –Efficiency-Access- Treatment
NPT: Normalization Process Theory
RCT: Randomized Controlled Trial
SafeTALK: Suicide Alertness For Everyone.
SMHFA: Scotland’s Mental Health First Aid
STORM: Skills Training On Risk Management
TIDieR: Template for Intervention Description and Replication
TTT: Train the Trainers
